# Implementing Anti-Racism Interventions in Healthcare Settings: A Scoping Review

**DOI:** 10.3390/ijerph18062993

**Published:** 2021-03-15

**Authors:** Nadha Hassen, Aisha Lofters, Sinit Michael, Amita Mall, Andrew D. Pinto, Julia Rackal

**Affiliations:** 1 Faculty of Environmental and Urban Change, York University, Toronto, ON M3J 1P3, Canada; nadha.hassen@utoronto.ca; 2 Women’s College Hospital, Toronto, ON M5S 1B2, Canada; aisha.lofters@wchospital.ca; 3 Department of Family and Community Medicine, Faculty of Medicine, University of Toronto, Toronto, ON M5G 1V7, Canada; andrew.pinto@utoronto.ca; 4 Knowledge Translation Program, Li Ka Shing Knowledge Institute, St. Michael’s Hospital, Toronto, ON M5B 1W8, Canada; sinit.michael@unityhealth.to; 5 Temerty Faculty of Medicine, University of Toronto, Toronto, ON M5S 1A8, Canada; a.mall@mail.utoronto.ca; 6 Dalla Lana School of Public Health, University of Toronto, Toronto, ON M5T 3M7, Canada; 7 Upstream Lab, MAP/Centre for Urban Health Solutions, Li Ka Shing Knowledge Institute, Unity Health Toronto, Toronto, ON M5B 1T8, Canada; 8 Department of Family and Community Medicine, St. Michael’s Hospital, Toronto, ON M5B 1W8, Canada

**Keywords:** anti-racism interventions, systemic racism, institutional racism, healthcare interventions, social determinants of health

## Abstract

Racism towards Black, Indigenous and people of colour continues to exist in the healthcare system. This leads to profound harm for people who use and work within these settings. This is a scoping review to identify anti-racism interventions in outpatient healthcare settings. Searching the peer-reviewed and grey literature, articles were screened for inclusion by at least two independent reviewers. Synthesizing the socio-ecological levels of interventions with inductively identifying themes, a conceptual model for implementing anti-racism interventions in healthcare settings is presented. In total, 37 peer-reviewed articles were included in the review, with 12 empirical studies and 25 theoretical or conceptual papers. Six grey literature documents were also included. Healthcare institutions need to incorporate an explicit, shared language of anti-racism. Anti-racism action should incorporate leadership buy-in and commitment with dedicated resources, support and funding; a multi-level approach beginning with policy and organizational interventions; transparent accountability mechanisms for sustainable change; long-term meaningful partnerships with Black, Indigenous, and people of colour (i.e., racialized communities); and ongoing, mandatory, tailored staff education and training. Decision-makers and staff in healthcare settings have a responsibility to take anti-racism action and may improve the success and sustainability of their efforts by incorporating the foundational principles and strategies identified in this paper.

## 1. Introduction

Racism and the racialization of Black, Indigenous and people of colour continues to be heavily present in the healthcare system, leading to profound harm for people who use and work within it. This systemic issue is particularly concerning considering the healthcare field’s commitment to health, wellness and doing no harm [1,2].

The literature documents numerous historical examples of racism in healthcare and in health research, including conducting medical experiments on and pathologizing the behaviours of Black people who were enslaved [3,4].Canada’s history includes nutrition experiments on Indigenous children, the sterilization of Indigenous women and the deportation of immigrants who were disabled [5,6].

This global history of colonialism and slavery has left a lasting impact on the delivery and utilization of healthcare today. Some contemporary examples of anti-Black racism in healthcare in North America include racial bias in pain assessment and treatment recommendations between White and Black patients based on false beliefs about biological differences [7], the prescription of significantly more analgesics to White patients compared to Black patients (74% versus 57%) in the emergency department for bone fractures [8], and the under-screening of Black Canadian women for cervical and breast cancer [9].

Experiences of racism and racialization (i.e., the process through which groups are socially constructed as different and unequal based on race) intersect with other aspects of identity and structural determinants of health, such as sexism, ageism, ableism, poverty, homophobia and transphobia, xenophobia and so on [10]. Marginalization and discrimination in healthcare also exist based on these other structural determinants of health. A study found that antipsychotic drugs were prescribed four times more often to children covered by Medicaid vs. private insurance, resulting in a disproportional impact on children of colour from lower-income households [11].

To date, the healthcare literature has focused on identifying and illustrating racial and ethnic disparities, often without naming the causes for those disparities [12]. There have been recent attempts to address racism in healthcare, including anti-Black and anti-Indigenous racism, by universities, healthcare institutions and non-profit organizations. Several interventions have been implemented and while there is growing interest in this work, to our knowledge no review and synthesis has been done to date. Our aim was to review and identify existing anti-racism interventions in healthcare settings and synthesize the key findings, challenges and unintended consequences of this necessary and challenging work.

## 2. Methods

**Figure 1:**
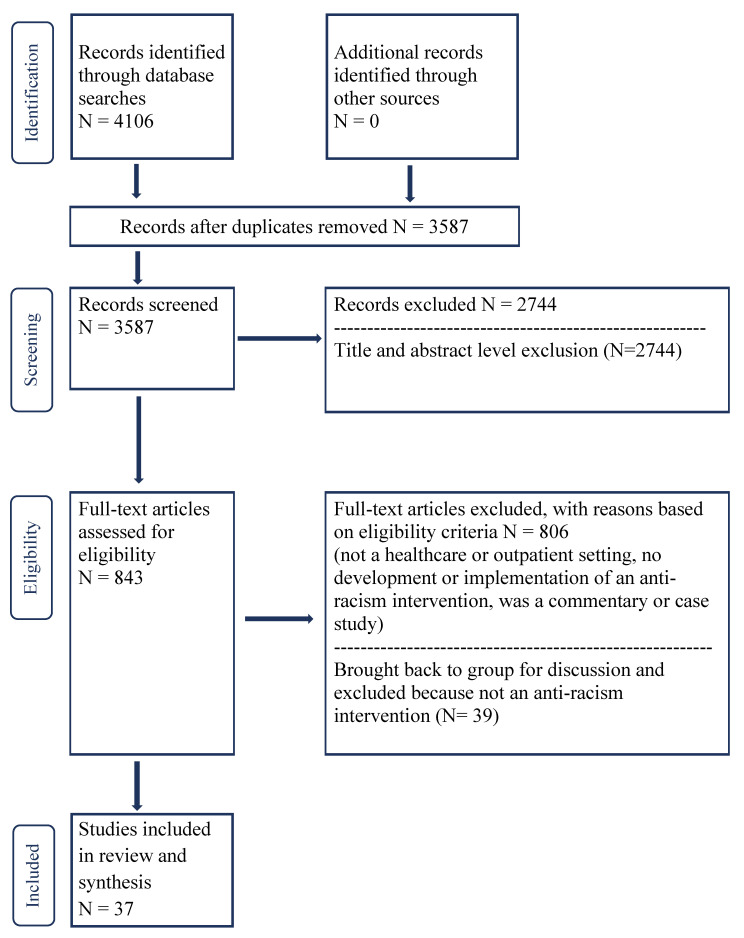
PRISMA flow diagram for peer-reviewed literature.

We followed Arksey and O’Malley’s framework [13] to conduct a rigorous scoping review and developed a search strategy in consultation with an information specialist. The databases searched included MEDLINE, CINAHL, EMBASE, Scopus and PsycINFO with key words including “race”, “racism”, “divers*”, “cultural competenc*”, resulting in 3587 citations after duplicates were removed [13]. The PRISMA flow diagram depicts the flow of information through the different phases (Figure 1. PRISMA flow diagram for peer-reviewed literature). The search strategy is available in the Supplementary Materials. A preliminary title and abstract review excluded articles beyond our stated scope. Each citation was reviewed by at least two reviewers and, if needed, was brought to the study team for further discussion. To be included, articles needed to focus on outpatients, healthcare providers providing care to outpatients or public health practitioners providing 1:1 care to individuals and to focus on the development or implementation of an anti-racism intervention (defined below).

We excluded purely educational settings, reviews, commentaries, news items and case studies. Each full-text article was reviewed by at least two team members and in the case of disagreement, was again brought back to the team for discussion. We extracted data on healthcare setting, healthcare provider and patient groups, anti-racism interventions, methods, findings, and frameworks. The quality assessment of articles was beyond the scope of this scoping review.

To identify relevant documents in the grey literature, we screened all eligible websites using the *Grey Matters* tool [14] for identifying international health-related grey literature, with the exception of drug and device regulatory approvals. The keywords used in the search were “race”, “racism”, “divers*”, “culture”, “cultural competency”, “equity”, “inclusion”. Of the 114 websites in the *Grey Matters* document, 28 websites were extracted as they included one or more of the keywords. A keyword search was then performed on each website, resulting in 78 individual web pages of which 41 met the inclusion criteria. Two reviewers (S.M., A.M.) examined each website and identified six grey literature documents for inclusion in the review.

## 3. Working Definitions of Anti-Racism Intervention and Types of Racism

We used Calliste and Dei’s [15] definition of an anti-racism intervention: an “action-oriented, educational and/or political strategy for systemic and political change that addresses issues of racism and interlocking systems of social oppression”. Anti-racism actions can come in many forms, including “individual transformation, organizational change, community change, movement-building, anti-discrimination legislation and racial equity policies in health, social, legal, economic and political institutions” [15]. This definition acknowledges the multiple ways that anti-racism action can occur and allowed us to include a wide range of intervention types.

Jones [16] describes three levels of racism: institutionalized racism, personally mediated racism and internalized racism. Institutionalized racism refers to “differential access to the goods, services, and opportunities of society by race” and is often normalized and legalized. Personally mediated racism refers to prejudice and discrimination between people where assumptions are made about people’s abilities, motives and actions based on their race, and assaults are made against people [16]. Internalized racism refers to the negative attitudes, beliefs and actions taken on by people of a racialized group about their own abilities and worth [16].

Additionally, the National Collaborating Centre for Determinants of Health defines systemic racism as “policies and practices within institutions such as regulations and standard ways of operating that lead to racially biased outcomes and experiences” [17].

Given the heterogeneity of the research, anti-racism interventions were also identified according to the level that the intervention targeted within a Social Ecological Model (SEM) [18]: individual, interpersonal, community, organizational and policy levels. Themes were inductively developed from the included articles and a conceptual model was iteratively developed, highlighting key strategies for anti-racism interventions in healthcare settings.

## 4. Results

### 4.1. Overview of the Literature

In total, 37 peer-reviewed articles were included in the review, with 12 empirical studies and 25 theoretical or conceptual papers. The empirical studies included the collection and analysis of data while theoretical or conceptual papers did not. Five conceptual papers described anti-racism interventions that were evaluated or in the process of being evaluated [19,20,21,22,23]. Table 1 summarizes the results of the peer-reviewed literature outlined below. Six grey literature documents were included [24,25,26,27,28,29]. A summary of the peer-reviewed articles and grey literature documents with detailed information and findings are presented in table format in Supplementary Materials.

Only 40% of articles explicitly defined racism or a specific type or level of racism, such as institutional racism or systemic racism. One third (32%) provided definitions for related terms, including stereotype, discrimination, prejudice, cultural safety, cultural competence/awareness/safety, racial and ethnic disparities, and unconscious bias. A quarter of the articles (26%) had no explicit definition for either racism or any related terms, although they used these terms within the articles. Those that did provide definitions of racism varied and were noted.

Of the peer-reviewed articles, 12 (32%) explicitly focused on anti-racism interventions for Indigenous populations as a patient group (includes Aboriginal and Torres Strait Islander people, Maori, First Nations, Inuit, and Metis/Native Americans) and only 5 (14%) explicitly focused on Black populations as a patient group (includes Black and African American) [30,31,32,52,53]. Twelve articles (32%) named other minority and racialized patient groups using terms like “minority groups”, “clients of colour”, “non-White”, “racial and ethnic minorities” and “socially stigmatized groups”.

There were a wide range of anti-racism interventions across different healthcare settings and provider groups. Almost half of the peer-reviewed articles focused on either nurses or physicians as healthcare provider groups (22% and 22%, respectively). Other articles focused on counsellors and psychologists (11%), social workers (3%) or pharmacists (3%), while 32% of articles did not specify a healthcare provider group. The anti-racism interventions were implemented across a range of settings, including hospitals (outpatients) (21%); network or regional level with direct patient reach (19%), such as the Henry Ford Health System in Michigan [33]; primary care (14%) and community-based settings providing outpatient care (11%), such as the NSW Health Education Centre Against Violence [54].

Anti-racism interventions were further stratified at differing socio-ecological levels. Table 2 outlines examples of anti-racism interventions in healthcare settings by intervention level.

*Individual-level interventions* included self-reflection tools, unconscious bias training and Implicit Association Tests that seek to make individuals aware of stereotypes about different racial groups that are unconsciously formed [30,34,35]. This type of intervention or training is targeted at individual transformation relating to knowledge, attitudes and behaviours. *Interpersonal-level interventions* focused on cultivating interactions (both formal and informal) between providers, patients and the provider–patient relationship that seek to address racial health disparities, mitigate harmful practices for racialized populations or address racist comments by clients [36]. For example, the Maori Practice Model (Te Kapunga Putohe) [59] was developed to integrate Maori practices into nursing practice and guide nurses in providing Maori-centred care to improve health outcomes for this population. *Community-level interventions* focused on developing meaningful relationships between the healthcare organization and populations that the healthcare setting serves or the geographic community that the organization is situated within. These interventions involved actively establishing ongoing, meaningful partnerships with racialized communities, including Black, Indigenous and other racialized groups to begin to address racism in healthcare [23,55,56]. Addressing institutional racism against Aboriginal people in hospitals needs committed physicians to engage, support, learn from and include Aboriginal community elders and members in discussion, planning and collaboration [56]. Investing time and resources in this process is an integral part of anti-racism work. *Organizational-level interventions* focused on structures and processes within an organization, including creating a consultation group, amending human resources policies, hosting workshops and conferences to effect organizational change [31,35,37,38,39,47,48,49,57]. Language translation and culturally- or linguistically-appropriate services were heralded (erroneously) as anti-racist interventions in some articles [40,41,42,49]. *Policy-level interventions* focused on policies, regulations, processes which include frameworks, policies, guidelines and recommendations at a system level [12,20,32,35,37,38,59]. For example, the National Health Service in the United Kingdom incorporated a workforce standard with indicators that compared metrics between “White and Black and minority ethnic” healthcare staff [53]. A policy statement with three principles was released by the American Society of Health-System Pharmacists to reduce racial and ethnic disparities [42]. These interventions are intended to provide broad-level mandates or guidance for uptake by stakeholders.

Many (62%) articles included interventions that were multi-level, i.e., targeted two or more levels. Most anti-racism interventions targeted the individual (54%), interpersonal (51%) and organizational (57%) levels. Only 21% of the peer-reviewed articles included an anti-racism intervention at the community-level and 24% included an intervention at the policy-level.

None of the included empirical studies targeted policy-level interventions and only two empirical studies included a community-level intervention [50,60]. Four of the six grey literature documents implemented policy-level interventions [26,27,28,29].

Some authors formed or identified recommendations, strategies, principles or lists of competencies for healthcare providers [21,35,37,43,48]. These articles often provided valuable insights but varied with respect to follow-up action.

Many articles focused on developing a new or original tool, training, workshop or curricula to varying degrees of success [19,20,23,30,31,33,34,44,45,49,51,56]. The evaluation of an *Undoing Racism* workshop [31] aimed at highlighting the role of racism in contributing to the Black–White gap in infant mortality found that the workshop was helpful in offering a common language and framework to discuss racism and identify changes to reduce disparities. However, results from 169 surveys noted that White respondents had fewer “likes” about the workshop compared to African Americans and were more likely to object to the perceived “confrontational style” of the workshop [31].

Bennet and Keating [52] identified key factors for effective race equity training, noting that “current approaches are fundamentally flawed”. These factors include whether the organization has a formal policy of anti-racism, established level of commitment, whether race equity training is voluntary or mandatory, the cost of attending, the (internal or external) facilitator’s qualifications, and whether the impact of the training is evaluated [52]. Continuous, ongoing training was considered better than one-time training [46]. Furthermore, training should avoid a “one size fits all” approach for staff which does not sufficiently address healthcare provider-specific issues [52].

There were challenges with engaging healthcare providers in the training process. Debriefing discussions of the interactive training tool *Privilege and Responsibility Curricular Exercise* [33] resulted in “divisive responses that facilitators had to challenge, such as denial, diversion, or anecdotal accounts questioning the existence of societal bias.” Anti-racism training needs to be ongoing, with the support of a skilled facilitator adept in this subject area [30]. Importantly, “an unintended consequence of training providers to avoid explicit bias is that their implicit bias is activated” [34]. One study identified that physicians “are the group least likely to attend” and make time for quality Aboriginal cultural orientation training [56]. Nurses were identified as key players and advocates in addressing inequities, establishing culturally safe and competent practices through transparent processes [48,55,59].

A mixed-methods study of a six-hour workshop on cultural competency with White, female occupational therapists found that study participants held significantly negative attitudes towards African Americans which were not ameliorated by the intervention [30]. The results demonstrated that participants held strong, persistent beliefs and racist attitudes towards Black people, were resistant to change despite evidence presented, and attributed any health disparities to a perceived “deficit” within that group. A key recommendation was to offer training in short sessions, spaced at least one week apart to allow for processing time by participants [30]. Papadopoulos, Tilki and Lees [21] present 15 principles to consider for effective cultural competency training.

### 4.2. Foundational Principles for Anti-Racism Interventions in Healthcare Settings

**Figure 2:**
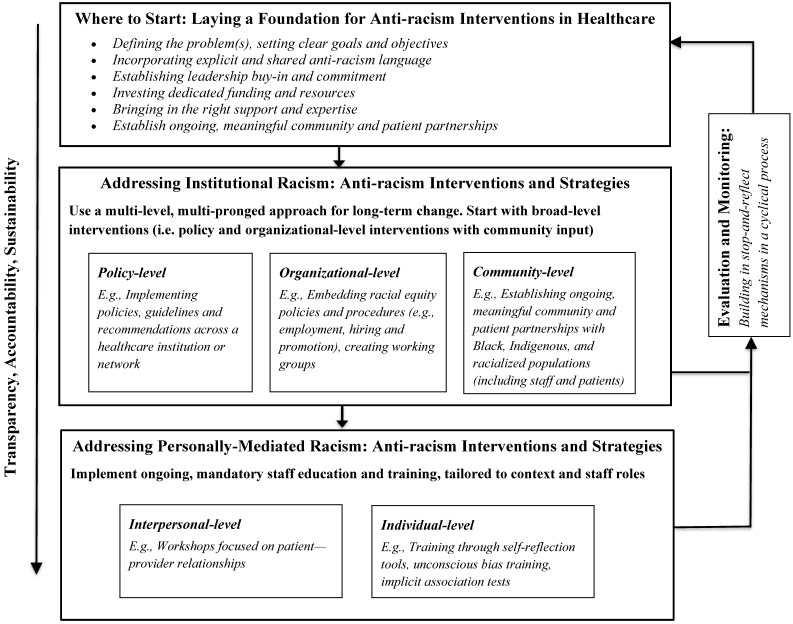
Overview of the principles and strategies for anti-racism interventions in healthcare settings.

The findings below present some key considerations for conducting anti-racism work in healthcare settings. These findings are summarized in the conceptual model (Figure 2. Overview of the principles and strategies for anti-racism interventions in healthcare settings.) The figure depicts the process, principles and strategies for consideration when implementing anti-racism interventions in healthcare settings.

### 4.3. Define the Problem(s) and Set Clear Goals and Objectives

Failing to clearly define a problem at the outset may result in interventions that are not aligned with intended goals, and consequently ineffective at achieving desired outcomes. Training and other initiatives must be tailored to address specific organizational needs and context [52] to tackle racism at various levels. For instance, a lack of knowledge and cultural competency around Indigenous health practices [57] is a specific problem with different objectives from reducing Black infant mortality [31]. Similarly, these objectives are distinct from trying to “build a culturally safe and respectful organisation that addresses individual and institutional racism” [23] or from implementing processes such as race-based data collection [38]. The literature underscores the need for thoughtful and intentional processes with respect to anti-racism work. Organizations ought to self-assess and “build organizational commitment to be inclusive, open, and progressive” while acknowledging power dynamics [48].

False assumptions may be made about what is needed within healthcare settings due to a lack of clarity and insufficient understanding and defining of the problem. As many articles focused on individual- or interpersonal-level interventions, such as training or workshops, it seems that there are assumptions that training will result in behaviour change and “solve” the problem of racism. One intervention found that, while general staff responses to training were positive, some employees were angry and struggled to accept the mandatory training, demonstrating “the complexity and depth of the racial issues and the importance of a multi-strategic approach that extends beyond the training itself” [23].

### 4.4. Incorporate Explicit and Shared Anti-Racism Language

There were a range of terms related to anti-racism that were used without a clear definition, or interchangeably with one another despite differences in meaning. Terms such as “cultural competency/awareness”, “diversity”, “inclusivity”, and “ethnic disparities” are broad and not explicitly defined in several of the articles [32,40,58,59,60]. The lack of explicit definitions within the literature and the tendency to use euphemistic language are problematic as this avoids explicitly naming racism [12,61]. This ambiguity risks undermining anti-racism action and interventions.

### 4.5. Establish Leadership Buy-In and Commitment

The Aboriginal Torres Strait Islander Strategic Leadership Committee in New South Wales [23] identified the foundational importance for strong, consistent visible leadership that addresses racism to build sustainable organizational change. Efforts should be made to facilitate meaningful involvement of leaders and governing bodies at different levels of an organization in antiracism initiatives [44]. The executive team need to be among the first to complete any training to demonstrate commitment, reinforce the importance of process and organizational change, and learn to provide informed support as staff undergo this process [23].

### 4.6. Invest Dedicated Funding and Resources

Several articles noted the need for dedicated funding for the implementation and evaluation of each anti-racism initiative [19,23,51,54,55,57]. The lack of funding to cover staff training was identified as one barrier to participation in training [51]. Leaders must commit to organizational investment and resources, including time, staff and funding for programs, services, training, and community participation e.g., with Indigenous communities [23].

### 4.7. Bring in the Right Support and Expertise

It can be valuable to convene a committee or group with the right expertise and lived experience and with clear objectives [12,22,23]. Subcommittees and working groups can “provide effective leadership and ongoing advice on health policy, planning, service delivery and resource allocation” [23]. An anti-racism initiative focusing on Aboriginal populations, ensured there were equal numbers of Aboriginal and non-Aboriginal committee members [23].

Negative consequences may be mitigated through bringing in the right support and expertise, such as consultants and facilitators with relevant anti-racism training and experience [30]. It is challenging to address and “deal with complex issues of racism and cultural differences and attempting to apply these to interpersonal relationships” [52]. As such, Steed [30] recommends involving members of racialized groups in the creation of interventions and educational materials and hiring skilled facilitators from specific racialized groups.

### 4.8. Establish Ongoing, Meaningful Community and Patient Partnerships

Several articles identified the importance of strong, meaningful relationships with racialized community and patient populations at multiple intervention levels [20,23,55,56]. Establishing effective partnerships with Indigenous populations is a long-term process that is complex [23]. Formal partnerships need to “engage Aboriginal staff and community representatives, including Elders and organizations, to ensure cultural appropriateness not just in planning but through all stages of development with regular communication” [23]. Interventions should be tailored “to address community-prioritized issues in order to develop a curriculum that is appropriate and effective” [44].

## 5. Anti-Racism Strategies for Implementation and Evaluation

### 5.1. Use a Multi-Level, Long-Term Approach

Several articles developed or identified frameworks that seek to take a multilevel, multi strategic approach to addressing racism in healthcare [12,20,23,38,50,54,55,57]. Anti-racism interventions are needed at systemic, organizational, interpersonal (patient⁄ provider) and patient- or family-centered levels simultaneously over an extended period of time to create impactful and sustainable change in healthcare settings [12,23,53,55]. Many organizations have focused on individual-level training; however, sustainability was a key issue and cannot be achieved through one-time interventions [55]. Griffith et al. [12] state that individual-level interventions should be implemented only after organizational-level interventions because “individual behaviour is shaped and promoted by organizational culture and practice”.

A key consideration for effective, sustainable anti-racist action is non-tokenistic action. Articles noted that organizations are “keen to be seen to be doing the right thing, meeting legal requirements and ‘ticking the boxes’” [52].

### 5.2. Embed Racial Equity Policies and Procedures (e.g., Hiring, Retention and Promotion)

Organizational and human resource policies, both internal and external, can contribute to racial health disparities without an explicit anti-racist lens [12,28,29,37]. When explicit anti-racist policies do not exist, these should be developed. The development of a Counter Racism Policy and Compliance Procedure, as part of a multistrategic effort to tackle racism within a regional health service in Australia, assisted with “the identification and reporting of racism in the workplace, with specific, executive-endorsed processes to investigate and address allegations of workplace racism when they arise” [23]. It was noted that “discrimination and inequities in the structure of policies, services and funding are readily identifiable but it is often the more subtle manifestations of racism throughout an organisation that go unrecognised” [23].

### 5.3. Link Mandatory Anti-Racism Work (Including Staff Education and Training) to Broader Systems of Power, Hierarchy and Dominance

Mandating anti-racism work within healthcare settings supports systemic change within institutions [29,31,44,52,54]. These initiatives should be linked to broader concepts and systems of oppression, including an understanding of power, hierarchy and dominance [12]. The aim is to address the root cause of racial health disparities, which is racism. To disrupt the status quo, training programs need to work towards systemic change, informed by critical theories [51]. A study found that healthcare providers believed that any system, such as healthcare, which is put into place by dominant culture should work equally well for everyone regardless of race, highlighting the need to tackle harmful misconceptions [30]. Anti-racist frameworks should also incorporate elements of power, oppression, community organizing principles and structural determinants of health (e.g., colonialism) [12].

### 5.4. Build in Stop-and-Reflect Mechanisms in a Cyclical Process

One of the key components in creating systems-level change to dismantle racism is “to increase infrastructure, accountability, and monitoring” [12]. The Dismantling Racism intervention, conducted with staff in a county public health department in the United States, deemed it necessary to inform and educate all staff, leadership and Board of Health about the philosophy, objectives, and accomplishments of mandatory training on an ongoing basis [44]. Not all interventions are necessarily good or effective at mitigating racism, signaling that appropriate monitoring and evaluation is necessary [12,52].

## 6. Discussion

We found some, but not many, examples of anti-racism interventions in outpatient healthcare settings in the literature. This work may not have been published publicly, either in the academic or grey literature. Those doing this critical work need to consider the opportunities for sharing their process and learnings on broader platforms for uptake and adaptation to local contexts. We do, however, recognize that the supports and resources to facilitate knowledge dissemination are often lacking. Few articles, especially those that were multi-level and long-term, included complete evaluation findings. Taking an implementation science perspective could be valuable to bridge the “know–do gap” [62].

Our review revealed limited literature on policies used to tackle anti-racism in healthcare settings, suggesting a gap in the implementation, uptake or evaluation of this type of intervention. There is a need to develop and evaluate anti-racism interventions that focus on organizational and policy-level change (as opposed to individual and interpersonal-levels) and these need to be supported through policy and dedicated funding [38]. Organizations should avoid further entrenching harmful dynamics and putting resources into trying to assess whether racism exists within their organizations (racism is pervasive and present everywhere).

This review highlights the opportunities and pitfalls of anti-racism interventions and the overemphasis on individual-level training. Organizations should avoid implementing standalone individual-level training and instead shift their focus and resources to policies and practices that seek to dismantle pervasive institutional and systemic racism through a multi-level approach. There are a number of valuable tools and resources for addressing organizational racism that exist outside of healthcare, within the education and non-profit sectors [63].

Language translation and linguistically appropriate services should be an integral part of access to healthcare services and are not necessarily innovative standalone anti-racism interventions. Healthcare organizations risk conflating services like language interpretation with interventions tackling racism at an institutional or systemic level [38].

It is imperative that authors explicitly name racism and define any terms used. The literature suggests that healthcare institutions are still grappling with using the word “anti-racism” in favour of less controversial terms, which risks watering down initiatives and skirting the important task of dismantling racism [52]. As less than half of the articles explicitly defined racism, it is challenging to glean the extent to which the articles take a critical anti-racist approach to tackling systemic and institutional racism as defined in this paper.

Ongoing collaborations are needed to tackle racism. Governing healthcare organizations, state and provincial health associations, and regulatory health bodies need to take leading roles in this work. In Canada, this includes the Canadian Medical Association, the College of Family Physicians of Canada and other similar bodies. In the United States, this work aligns with and builds on legislation like the 1964 Civil Rights Act and more recently, the 2010 Patient Protection and Affordable Care Act. The Affordable Care Act included provisions to ensure that federal data collection efforts included data on race, ethnicity, sex, primary language and disability status to highlight disparities discriminating against African American populations. The conversation on collecting race-based data is polarized as, given historical events, this process can be unsafe for and exploitative of certain groups [64]. Race-based data collection initiatives need to be more than simply a checkbox act of virtue signaling and needs to be undertaken in collaboration with racialized communities with the objectives and applications for the data made explicitly clear.

After the Civil Rights Act of 1964 in the United States, liberal discourses have ushered in a sense of a post-racial world, which has created a “new racialism” [65] that we have yet to critically analyze and address in healthcare. As a result, anti-Blackness, anti-Indigeneity, and racism against other people of colour is pervasive across healthcare settings, and until recently has often gone unchallenged. Given the current global context, with the call from Black Lives Matter protests worldwide, the recognition of racism against Indigenous peoples, and rising racism and xenophobia against people of colour, race-related discussions need to be centered around systems of oppression and their intersections.

## 7. Conclusions

Decision-makers in healthcare institutions have a responsibility to take anti-racism action [66]. In this paper, we present key processes, principles and strategies for consideration when anti-racism interventions are planned and executed at various levels in healthcare. This includes ensuring leadership includes Black, Indigenous and racialized members, auditing and implementing policies and process to ensure racism is addressed, and creating transparent accountability mechanisms and processes that are communicated to all. This information will help guide organizations as they facilitate difficult conversations, such as who is benefitting from certain initiatives. Anti-racism interventions will need to be tailored to the communities being served by healthcare settings, and this cannot be done without centering the voices and experiences of racialized staff, patients and communities.

Healthcare institutions need to reflect critically on whether they are ready to make the commitment necessary to do this work and invest time and money in the process to bring about sustainable system-level change, or else consider not doing it at all. Without a solid commitment to incorporating the principles of anti-racism work developed by racialized people, primarily Black and Indigenous scholars and activists, over decades, healthcare institutions will continue to create and perpetuate harm.

There is potential for synergies between healthcare, public health and other fields. Healthcare has a responsibility to learn from and collaborate with people from the social sciences and humanities, including critical theorists, sociologists, philosophers, historians and other disciplines to begin to heal decades of systemic racism. Given the relative paucity of data on what types of anti-racism interventions are effective in changing the experience of racism in healthcare settings, we urge healthcare institutions embarking on this work to evaluate their interventions for both processes and outcomes to contribute to the evidence base. The challenges encountered, not just the successes, will critically inform this work. Future research and evaluation of anti-racism interventions in healthcare are necessary, especially at the policy and community levels.

## Figures and Tables

**Table 1:** Summary of results from peer-reviewed literature (*n* = 37)**.**

Country	Number of Articles (%)
United States of America [12,19,30,31,32,33,34,35,36,37,38,39,40,41,42,43,44,45,46]	19 (51%)
Canada [47,48,49,50,51]	5 (14%)
United Kingdom [21,22,52,53]	4 (11%)
Australia [23,54,55,56,57,58]	6 (16%)
New Zealand [20,59,60]	3 (8%)
**Target Healthcare Provider Group**	
Nurses [41,43,47,48,55,58,59,60]	8 (22%)
Physicians [19,32,34,35,38,40,56,57]	8 (22%)
Psychologists/Counsellors [21,36,45,46,52]	5 (11%)
Social Workers [54]	1 (3%)
Occupational Therapists [30]	1 (3%)
Pharmacists [42]	1 (3%)
Other/Not specified	13 (35%)
**Target Patient Group**	
Indigenous populations as a patient group (includes Aboriginal and Torres Strait Islander people, Maori, First Nations, Inuit, and Metis/Native Americans) [20,23,26,47,50,51,54,55,56,57,58,59,60]	12 (32%)
Black populations as a patient group (includes Black and African American) [30,31,32,52,53]	5 (14%)
Other minority and racialized patient groups using terms like “minority groups”, “clients of colour”, “non-White”, “racial and ethnic minorities”	12 (32%)
**Healthcare Setting**	
Hospitals (outpatients) [22,35,37,47,51,55,56]	8 (21%)
Network or regional level with direct patient reach [12,20,23,33,39,42,44]	7(19%)
Primary care [21,43,46,50,58,60]	6 (14%)
Community-based settings providing outpatient care [19,30,31,48,52,54]	6(14%)
**Level of Intervention**	
Individual [19,21,22,30,34,37,38,40,41,42,47,50,54,55,56,58,59]	20 (54%)
Interpersonal [20,23,30,33,36,37,38,41,43,44,45,47,50,51,52,54,56,58,59]	19 (51%)
Community [12,23,48,50,55,56,59,60]	8 (21%)
Organizational [12,22,31,32,35,37,38,39,42,44,47,48,49,51,52,53,54,55,56,57]	21 (57%)
Policy [12,20,23,32,35,37,38,42,53]	9 (24%)

**Table 2:** Examples of anti-racism interventions in healthcare settings by intervention-level.

**Individual-level: 20 articles (54%)**	- Deliver cultural competency training on providing culturally competent care (addressing concepts related to racism, unconscious or implicit bias, stereotype, prejudice) [22,30,34,37,38,42,46] (e.g., 33-h cultural medicine curriculum within residency program [19]). - Provide continuous, ongoing training with an explicit anti-racism focus [46,52]. - Incorporate critical reflection on knowledge, attitudes, beliefs and practice/reflexivity [46,55,56,58].
**Interpersonal-level: 19 articles (51%)**	- Provide workshops for healthcare providers focusing on privilege and cultural competency training [20,23,30,33], the impact on clinical decision-making [37,38], on cultural schemas [45] and other strategies like appropriate humour [51] and relational accountability [58]. - Incorporate reflective questions for cultural safe healthcare [56]. - Develop and implement guidelines on how to address racist or prejudicial comments in psychotherapy [36].
**Community-level: 8 articles (21%)**	- Develop ongoing, meaningful partnerships with Aboriginal/Indigenous stakeholders and communities [23] and with Aboriginal/Indigenous Elders [56]. - Actively engage Aboriginal/Indigenous and racialized communities at multiple levels and throughout the process [55,56,59]. - Reorganize power by strengthening community relationships and forming caucus groups for anti-racist community organizing [12].
**Organizational-level: 21 articles (57%)**	- Develop a strategic leadership committee, consultation group, team (“charged with monitoring and addressing policies and practices, resource allocations, relational structures, organizational norms and values, and individual skills and attitudes”) [12] and implementing action plans that work towards anti-racist strategic goals [12,22,23,42]. - Have commitment from leadership in organizational investment [12,53]. - Ensure core leadership support that articulates diversity as a high institutional priority and organizational investment in supportive communication to all relevant stakeholders [53]. - Implement counter-racism policy compliance procedures [22,23]. - Build supports for Indigenous and racialized staff [55,56] (e.g., Indigenous hospital liaison officers [57]). - Identify and improve culturally unsafe systems and improve hospital and primary healthcare links [55]. - Collect data to identify racial disparities and their sources [37,38] (e.g., race-based data collection [37,38,42]). - Educate healthcare providers on anti-racism through several venues, grand rounds, newsletters, public relations campaigns, ongoing curricula, workshops [37,38,51] and provide ongoing orientation for new healthcare providers [57]. - Incorporate anti-racism into quality improvement initiatives [37,38] (e.g., indicators for the workforce race equality standard [53]).
**Policy-level: 9 articles (24%)**	- Recruit, retain, and promote Black, Indigenous and people of colour at all levels of the academic ladder in mainstream admission and promotion policy [12,32,35,37,38] and in the healthcare workforce [32,42]. - Increase infrastructure, accountability, transparency and monitoring [12,23,55]. - Increase Maori/Indigenous participation and partnership in decision making through shared leadership in policymaking (e.g., use Maori/Indigenous models of health in policymaking) [20,23]. - Mandate targets and actions [53]. - Implement multiple strategies at policy, organizational, community, interpersonal, and individual levels simultaneously over a long period [53].

## Data Availability

Not applicable.

## Supplementary Materials

The following are available online at https://www.mdpi.com/1660-4601/18/6/2993/s1.
