# Supplementary material for: Implementing Anti-Racism Interventions in Healthcare Settings: A Scoping Review
Source: Int J Environ Res Public Health. 2021 Mar 15;18(6):2993. doi: 10.3390/ijerph18062993 (PMC8000324; doi:10.3390/ijerph18062993)
Supplement: Supplementary file 1 [file ijerph-18-02993-s001.zip › Appendix B_Grey Literature_Nov202020.docx]

**Appendix B: Grey literature documents included in the review**

| **Organization** | **The Hospital for Sick Children (SickKids) ^24^** | **Registered Nurses’ Association of Ontario (RNAO) ^25^** | **Public Health England (PHE) ^26^** | **National Collaborating Centre for Determinants of Health (NCCDH) ^27^** | **North Western Health Board (NWHB) ^28^** | **Health Improvement Scotland**  **(HIS) ^29^** |
| --- | --- | --- | --- | --- | --- | --- |
| **Type of anti-racism intervention** | Modules | Policy statement | Evidence summary and recommendations | Collection of tools and resources | Code of practice | Screening Checklist |
| **Target Audience** | Healthcare providers | Registered nurses in Ontario | “government, local government, the NHS, Parliament, industry” | Any Canadian Health Practitioner | Health services in North West Ireland | Healthcare facilities |
| **Objective** | The aim of this evaluation is to gather healthcare providers’ opinions of the e-learning series and to assess the impact of cultural competence education on healthcare providers’ knowledge, skills and practice | Describing the organization’s position against racism | “to inform local and national action by PHE and other bodies” | “... to understand and act against structural racism” | Their goal is to ensure equality of access, participation and outcome for all who avail of their services, participate in their programs and / or work with them as employees | The aim of this policy is to communicate Healthcare Improvement Scotland (HIS) commitment to the promotion of equality |
| **Definition of racism** | No explicit definition | Racism has the effect of excluding groups of people (based on their race, colour, nationality, ethnic or ethno- religious origin) from decision- making processes and leadership and economic opportunities. Racism is both an attitude as well as the specific actions resulting from that attitude. The effect is to marginalize and oppress some people and to sustain advantages for people of certain social groups | Institutional Racism – defined by Macpherson as ‘… the collective failure of an organisation to provide an appropriate or professional service to people because of their colour, culture or ethnic origin. It can be seen or detected in processes, attitudes and behaviour which amount to discrimination through unwitting prejudice, ignorance, ignorance, thoughtlessness and racist stereotyping, which disadvantage minority ethnic people. (Reference: Home Office. The Stephen Lawrence Inquiry. Report of an Inquiry by Sir William Macpherson of Cluny. 1999. London: Stationery Office) | No explicit definition | Racism is a specific form of discrimination and exclusion experienced by Black people, people of colour and other recognized minority ethnic groups such as travellers, as a result of their skin colour, nationality, ethnic or cultural background. | No explicit definition |
| **Are any possible targets of racism specifically identified?** | Unsure – modules no longer available | No | Yes; “Black”, “Asian”, “Gypsy”; several other specific countries of origin | Yes; “Indigenous and racialized” groups | Yes; Black people, people of colour and other recognized minority ethnic groups | No |
| **Is the material mandatory for the target audience?** | No | No | No | No | No | No |
| **Are specific examples of anti-racist interventions or strategies given?** | No | No | Yes | No | No | No |
