# Supplementary material for: Implementing Anti-Racism Interventions in Healthcare Settings: A Scoping Review
Source: Int J Environ Res Public Health. 2021 Mar 15;18(6):2993. doi: 10.3390/ijerph18062993 (PMC8000324; doi:10.3390/ijerph18062993)
Supplement: Supplementary file 1 [file ijerph-18-02993-s001.zip › Search strategy_Anti-R Interventions_Jan2021.doc]

On 25 April 2018, the Health Sciences Library at St. Michael's Hospital conducted a literature search to identify interventions to identify racism in outpatient settings. The PICO was identified as:

**Population**—outpatients experiencing racism. This included outpatient care, issues affecting outpatients, and health facilities that serve them (lines 1 through 27) combined with the terms racism, bias, and associated terms (28-50) and populations (51-70)

**Intervention**—Interventions, goals, and desired outcomes (72-97)

**Comparison—none**

**Outcome**—to be determined by systematic analysis

The search was rerun in leading health sciences databases (CINAHL, the Cochrane Databases, Embase and PsychInfo) adapted from the original Medline search. A combined total of 4106 citations were collected. Once duplicates were removed, 3587 citations were submitted to the research team for title and abstract review.

****Health Sciences Databases****

**Database: All Ovid Medline <1946 - present>**

**Search Strategy:**

--------------------------------------------------------------------------------

1	exp OUTPATIENTS/ [Primary population] (13425)

2	outpatient*.ti,ab,tw. (144968)

3	exp Ambulatory Care/ [MeSH associated with outpatients] (49876)

4	exp Health Services, Indigenous/ (2752)

5	(Health Services adj5 Indigenous).ti,ab,kf. (258)

6	exp General Practice/ (71792)

7	((family or general) adj2 (health* or practice)).ti,ab,tw. (81551)

8	exp Primary Health Care/ (136782)

9	exp Urban Health/ (17059)

10	exp Health Knowledge, Attitudes, Practice/ [factors or circumstances affecting outpatients] (95443)

11	exp Health Services Accessibility/ (99557)

12	((access adj5 healthcare) or (access adj5 health care)).ti,ab,kf. (15823)

13	exp attitude to health/ (369028)

14	health knowledge.ti,ab,kf. (2655)

15	exp "Social Determinants of Health"/ (1489)

16	exp "Attitude of Health Personnel"/ (144141)

17	(Attitude adj5 Health Personnel).ti,ab,kf. (212)

18	outpatient department/ [locations associated with outpatients] (0)

19	(health center* or health centre*).ti,ab,kf. (26546)

20	exp Maternal-Child Health Centers/ (2271)

21	exp Community Mental Health Centers/ (3102)

22	exp Maternal-Child Health Centers/ (2271)

23	exp pain clinic/ (1349)

24	substance abuse treatment centers/ (5021)

25	(walkin or walk-in or urgent care).ti,ab,kf. (3734)

26	or/1-25 (972595)

27	exp racism/ (1441)

28	exp racism/ [not Prejudice] (1441)

29	racism.ti,ab,kf. (2544)

30	(white extreme* or white suprem*).ti,ab,kw. (31)

31	27 or 28 or 29 or 30 (3672)

32	(anti-arab or anti-black or anti-aboriginal or anti-indigenous or anti-brown or anti-asian or xenophob*).ti,ab,kf. (191)

33	(anti-semit* or anti-Jewish).ti,ab,kf. (191)

34	(Islamophob* or anti-muslim).ti,ab,kf. (35)

35	(anti-sikh* or anti-hindu*).ti,ab,kf. (0)

36	apartheid.ti,ab,kf. (562)

37	(bias adj3 race).ti,ab,tw. (157)

38	(bias adj3 racial).ti,ab,kf. (429)

39	(bigotry or bigot*).ti,ab,kf. (147)

40	exp COLONIALISM/ (1309)

41	colonial*.ti,ab,kf. (5435)

42	exp desegregation/ (2)

43	(segregation adj5 (race or racial)).mp. [mp=title, abstract, original title, name of substance word, subject heading word, keyword heading word, protocol supplementary concept word, rare disease supplementary concept word, unique identifier, synonyms] (383)

44	exp prejudice/ [includes exp racism, but also identifies other forms of discrimination] (26850)

45	(religious hatred or religious intolerance).ti,ab,tw,kf. (8)

46	social marginali*.ti,ab,kf. (207)

47	exp social stigma/ (5247)

48	exp social desirability/ (4247)

49	exp stereotyping/ (10108)

50	or/32-48 (42798)

51	("People of color" or "people of colour").ti,ab,kf. (385)

52	(African or afro*).ti,ab,kf. (124553)

53	exp african continental ancestry group/ (79835)

54	exp American Native Continental Ancestry Group/ (20057)

55	Asian Continental Ancestry Group/ (55666)

56	exp oceanic ancestry group/ (8995)

57	(metis or mestizo).ti,ab,kf. (1211)

58	african*.mp. or afro*.ti,ab,kf. [mp=title, abstract, original title, name of substance word, subject heading word, keyword heading word, protocol supplementary concept word, rare disease supplementary concept word, unique identifier, synonyms] (180052)

59	(aboriginal or eskimo* or Inuit* or Inuk or Metis* or first nation* or indigenous Canadians or native Canadian or Native America or Maori* or pacific islander* or American indian* or Amerindian or native alaska* or Alaska native or native Hawaiian or Torres strait or on-reserve or off-reserve or tribal or autochton* or amerindian* or indigene*).ti,ab,kf. (31084)

60	(arab* or persian*).ti,ab. (114115)

61	exp Jews/ or semitic peoples.ti,ab,kf. (1)

62	exp Asian/ (62331)

63	Asian*.ti,ab,kf. (70865)

64	exp Hispanic Americans/ (28434)

65	hispanic.ti,ab. (32488)

66	exp Roma/ (787)

67	Roma*.ti,ab,kf. (18460)

68	or/51-67 (482990)

69	50 and 68 (7042)

70	31 or 69 (9270)

71	26 and 70 (2114)

72	exp cultural competence/ [interventions] (4466)

73	cultural competenc:.ti,ab,kf. (2960)

74	exp Culturally Competent Care/ (733)

75	exp cultural diversity/ (10534)

76	exp Capacity Building/ (1736)

77	exp Community Participation/ (37803)

78	(community engag* or community particip* or community involv*).ti,ab,kf. (7432)

79	exp Evaluation Studies/ (234174)

80	exp Practice Guideline/ (23861)

81	guideline.ti,ab,kf. (44187)

82	exp Health Services Research/ (150572)

83	in service training.ti,ab,kf. (1053)

84	intervention.ti,ab,kf. (495864)

85	intersectionality.mp. (432)

86	knowledge transfer.ti,ab,kf. (1319)

87	exp LEADERSHIP/ (36894)

88	needs assessment/ (27118)

89	exp personnel management/ (143893)

90	exp Staff Development/ (8602)

91	((professional or staff) adj2 development).ti,ab,tw. (9760)

92	racism awareness.ti,ab,kf. (2)

93	exp program evaluation/ (67993)

94	(policy or polic*).ti,ab,kf. (232704)

95	((policy or practice) adj5 equitable).ti,ab,kf. (143)

96	exp Quality Assurance, Health Care/ or exp Quality Improvement/ or exp Total Quality Management/ (314572)

97	quality improvement.ti,ab,kf. (28499)

98	or/72-97 (1594079)

99	71 and 98 (929)

100	remove duplicates from 99 (925)

101	limit 100 to (addresses or autobiography or bibliography or biography) (4)

102	limit 100 to comment (19)

103	limit 100 to editorial (12)

104	limit 100 to letter (13)

105	102 or 103 or 104 or 105 (33)

106	100 not 106 (892)

***************************

**CINAHL: 309**

| # | Query | Results | |  |
| --- | --- | --- | --- | --- |
| S39 | S7 AND S24 AND S38 | | 309 |  |
| S38 | S26 OR S27 OR S28 OR S29 OR S30 OR S31 OR S32 OR S33 OR S34 OR S35 OR S36 OR S37 | | 667,748 |  |
| S37 | (MH "Quality Improvement+") OR "quality improvement" OR (MH "Quality Assessment+") OR (MH "Quality of Health Care+") | | 590,929 |  |
| S36 | (MH "Staff Development+") OR "staff development" | | 29,907 |  |
| S35 | (MM "Leadership") | | 18,223 |  |
| S34 | "knowledge transfer" | | 627 |  |
| S33 | "intersectionality" | | 337 |  |
| S32 | (MM "Transcultural Nursing") OR "transcultural nursing" | | 3,266 |  |
| S31 | (MM "Education, Continuing (Credit)") OR (MH "Education, Continuing+") | | 33,412 |  |
| S30 | "community involve" | | 3 |  |
| S29 | "community engage*" | | 1,173 |  |
| S28 | "community participation" | | 2,073 |  |
| S27 | "capacity building" | | 1,623 |  |
| S26 | (MM "Cultural Competence") | | 4,204 |  |
| S25 | S7 AND S24 | | 1,838 |  |
| S24 | S8 OR S9 OR S10 OR S11 OR S12 OR S13 OR S14 OR S15 OR S16 OR S17 OR S18 OR S19 OR S20 OR S21 OR S22 OR S23 | | 42,549 |  |
| S23 | "sterotyping" | | 2 |  |
| S22 | "xenophobia" | | 38 |  |
| S21 | "segregation" | | 1,331 |  |
| S20 | "colonialism" | | 148 |  |
| S19 | "race bias" OR (MH "Race Relations+") | | 5,385 |  |
| S18 | (MM "Cultural Bias") OR "racial bias" | | 2,426 |  |
| S17 | "anti-semit*" | | 23 |  |
| S16 | "anti-asian" | | 1 |  |
| S15 | "anti-indigenous" | | 1 |  |
| S14 | "anti-aboriginal" | | 0 |  |
| S13 | "anti-arab" | | 2 |  |
| S12 | "anti black" | | 11 |  |
| S11 | (MH "Discrimination+") OR "discrimination" | | 32,503 |  |
| S10 | "white supremacy" | | 13 |  |
| S9 | (MM "Prejudice") OR "prejudice" OR (MM "Stigma") | | 13,285 |  |
| S8 | (MM "Racism") OR "racism" OR (MM "Cultural Bias") | | 5,567 |  |
| S7 | S1 OR S2 OR S3 OR S4 OR S5 OR S6 | | 180,887 |  |
| S6 | (MM "Rural Health Centers") OR (MH "Community Health Centers+") OR (MH "Rehabilitation Centers+") OR "health centers" | | 15,016 |  |
| S5 | (MM "Urban Health") OR (MM "Urban Health Services") OR (MM "Hospitals, Urban") OR (MM "Attitude of Health Personnel") OR (MM "Pe  Ssonnel, Health Facility") OR (MH "Health Services Needs and Demand+") | | 42,860 |  |
| S4 | (MM "Primary Health Care") OR "Primary Health Care" OR (MH "Health Services Needs and Demand+") | | 72,744 |  |
| S3 | (MM "Family Practice") OR "General Practice" | | 20,070 |  |
| S2 | (MM "Health Services, Indigenous") OR "Health Services, Indigenous" OR (MH "Health Services Misuse+") OR (MM "Indigenous Health") | | 6,384 |  |
| S1 | (MM "Outpatients") OR "OUTPATIENTS" OR (MM "Outpatient Service") OR (MM "Ambulatory Surgery") OR (MM "Ambulatory Care Facilities") | |  | |

**Database: EBM Reviews - Cochrane Database of Systematic Reviews <2005 to April 25, 2018>, EBM Reviews - ACP Journal Club <1991 to April 2018>, EBM Reviews - Database of Abstracts of Reviews of Effects <1st Quarter 2016>, EBM Reviews - Cochrane Clinical Answers <March 2018>, EBM Reviews - Cochrane Central Register of Controlled Trials <March 2018>, EBM Reviews - Cochrane Methodology Register <3rd Quarter 2012>, EBM Reviews - Health Technology Assessment <4th Quarter 2016>, EBM Reviews - NHS Economic Evaluation Database <1st Quarter 2016>**

**Search Strategy:**

--------------------------------------------------------------------------------

1	exp OUTPATIENTS/ [Primary population] (1163)

2	outpatient*.ti,ab,tw. (28094)

3	exp Ambulatory Care/ [MeSH associated with outpatients] (3746)

4	exp Health Services, Indigenous/ (30)

5	(Health Services adj5 Indigenous).ti,ab,kf. (5)

6	exp General Practice/ (2493)

7	((family or general) adj2 (health* or practice)).ti,ab,tw. (11214)

8	exp Primary Health Care/ (4970)

9	exp Urban Health/ (356)

10	exp Health Knowledge, Attitudes, Practice/ [factors or circumstances affecting outpatients] (5176)

11	exp Health Services Accessibility/ (1013)

12	((access adj5 healthcare) or (access adj5 health care)).ti,ab,kf. (444)

13	exp attitude to health/ (30820)

14	health knowledge.ti,ab,kf. (273)

15	exp "Social Determinants of Health"/ (14)

16	exp "Attitude of Health Personnel"/ (2108)

17	(Attitude adj5 Health Personnel).ti,ab,kf. (1)

18	outpatient department/ [locations associated with outpatients] (0)

19	(health center* or health centre*).ti,ab,kf. (2238)

20	exp Maternal-Child Health Centers/ (45)

21	exp Community Mental Health Centers/ (114)

22	exp Maternal-Child Health Centers/ (45)

23	exp pain clinic/ (79)

24	substance abuse treatment centers/ (361)

25	(walkin or walk-in or urgent care).ti,ab,kf. (6221)

26	or/1-25 (81300)

27	exp racism/ (27)

28	exp racism/ [not Prejudice] (27)

29	racism.ti,ab,kf. (30)

30	(white extrem* or white suprem*).ti,ab,kw. (2)

31	27 or 28 or 29 or 30 (55)

32	(anti-arab or anti-black or anti-aboriginal or anti-indigenous or anti-brown or anti-asian or xenophob*).ti,ab,kf. (2)

33	(anti-semit* or anti-Jewish).ti,ab,kf. (0)

34	(Islamophob* or anti-muslim).ti,ab,kf. (0)

35	(anti-sikh* or anti-hindu*).ti,ab,kf. (0)

36	apartheid.ti,ab,kf. (1)

37	(bias adj3 race).ti,ab,tw. (12)

38	(bias adj3 racial).ti,ab,kf. (26)

39	(bigotry or bigot*).ti,ab,kf. (1)

40	exp COLONIALISM/ (0)

41	colonial*.ti,ab,kf. (10)

42	exp desegregation/ (47)

43	(segregation adj5 (race or racial)).mp. [mp=ti, ab, tx, kw, ct, ot, sh, hw] (6)

44	exp prejudice/ [includes exp racism, but also identifies other forms of discrimination] (343)

45	(religious hatred or religious intolerance).ti,ab,tw,kf. (0)

46	social marginali*.ti,ab,kf. (4)

47	exp social stigma/ (143)

48	exp social desirability/ (181)

49	exp stereotyping/ (342)

50	or/32-48 (683)

51	("People of color" or "people of colour").ti,ab,kf. (10)

52	(African or afro*).ti,ab,kf. (7161)

53	exp african continental ancestry group/ (2717)

54	exp American Native Continental Ancestry Group/ (277)

55	Asian Continental Ancestry Group/ (1937)

56	exp oceanic ancestry group/ (154)

57	(metis or mestizo).ti,ab,kf. (25)

58	african*.mp. or afro*.ti,ab,kf. [mp=ti, ab, tx, kw, ct, ot, sh, hw] (9753)

59	(aboriginal or eskimo* or Inuit* or Inuk or Metis* or first nation* or indigenous Canadians or native Canadian or Native America or Maori* or pacific islander* or American indian* or Amerindian or native alaska* or Alaska native or native Hawaiian or Torres strait or on-reserve or off-reserve or tribal or autochton* or amerindian* or indigene*).ti,ab,kf. (3320)

60	(arab* or persian*).ti,ab. (2213)

61	semitic peoples.ti,ab,kf. (0)

62	exp Asian/ (2124)

63	Asian*.ti,ab,kf. (4427)

64	exp Hispanic Americans/ (1220)

65	hispanic.ti,ab. (2641)

66	exp Roma/ (0)

67	Roma*.ti,ab,kf. (586)

68	or/51-67 (23645)

69	50 and 68 (124)

70	31 or 69 (153)

71	26 and 70 (45)

72	exp cultural competence/ [interventions] (141)

73	cultural competenc:.ti,ab,kf. (44)

74	exp Culturally Competent Care/ (0)

75	exp cultural diversity/ (72)

76	exp Capacity Building/ (24)

77	exp Community Participation/ (1143)

78	(community engage* or community particip* or community involve*).ti,ab,kf. (514)

79	exp Evaluation Studies/ (823)

80	exp Evaluation Studies/ (823)

81	exp Practice Guideline/ (0)

82	guideline.ti,ab,kf. (3733)

83	exp Health Services Research/ (2360)

84	in service training.ti,ab,kf. (71)

85	intervention.ti,ab,kf. (127382)

86	intersectionality.mp. (4)

87	knowledge transfer.ti,ab,kf. (82)

88	exp LEADERSHIP/ (171)

89	needs assessment/ (360)

90	exp personnel management/ (2138)

91	exp Staff Development/ (81)

92	((professional or staff) adj2 development).ti,ab,tw. (328)

93	racism awareness.ti,ab,kf. (0)

94	exp program evaluation/ (5762)

95	(policy or polic*).ti,ab,kf. (6919)

96	((policy or practice) adj5 equitable).ti,ab,kf. (3)

97	exp Quality Assurance, Health Care/ or exp Quality Improvement/ or exp Total Quality Management/ (3637)

98	quality improvement.ti,ab,kf. (1543)

99	or/72-98 (142600)

100	71 and 99 (22)

***************************

**Database: Embase Classic+Embase <1947 to 2018 April 27>**

**Search Strategy:**

--------------------------------------------------------------------------------

1	exp OUTPATIENTS/ [Primary population] (103102)

2	outpatient*.ti,ab,tw. (231186)

3	exp Ambulatory Care/ [types of outpatient care] (46386)

4	exp indigenous health care/ (510)

5	(Health Services adj5 Indigenous).ti,ab,tw. (138)

6	exp general practice/ (79922)

7	((family or general) adj2 (health* or practice)).ti,ab,tw. (101906)

8	exp Primary Health Care/ (145602)

9	exp urban health/ (407)

10	exp Health Knowledge, Attitudes, Practice/ [factors or circumstances affecting outpatients] (100824)

11	((access adj5 healthcare) or (access adj5 health care)).ti,ab,tw. (18602)

12	exp attitude to health/ (100824)

13	health knowledge.ti,ab,tw. (2690)

14	exp "social determinants of health"/ (3794)

15	(Attitude adj5 Health Personnel).ti,ab,tw. (39)

16	outpatient department/ [Health care facility locations associated with outpatients] (73587)

17	exp health center/ (30819)

18	exp community mental health center/ (3534)

19	maternal-child health centers/ (580)

20	exp pain clinic/ (3086)

21	exp drug dependence treatment/ (19924)

22	(health center or health centre).ti,ab,tw. (18349)

23	(walkin or walk-in).ti,ab,tw. (2945)

24	urgent care.ti,ab,tw. (2432)

25	1 or 2 or 3 or 4 or 5 or 6 or 7 or 8 or 9 or 10 or 11 or 12 or 13 or 14 or 15 or 16 or 17 or 18 or 19 or 20 or 21 or 22 or 23 or 24 (760524)

26	exp racism/ [primary Issue] (4170)

27	exp race relation/ (4648)

28	racism.ti,ab,tw. (2676)

29	(white extreme* or white suprem*).ti,ab,kw. (23)

30	26 or 27 or 28 or 29 (6333)

31	(anti-arab or anti-black or anti-aboriginal or anti-indigenous or anti-brown or anti-asian or xenophob*).ti,ab,tw. (194)

32	(anti-semit* or anti-Jewish).ti,ab. (189)

33	(Islamophob* or anti-muslim).ti,ab,tw. (33)

34	(anti-sikh* or anti-hindu*).ti,ab,tw. (0)

35	apartheid.ti,ab,tw. (541)

36	(bias adj3 ethnic).ti,ab,tw. (161)

37	(bias adj3 race).ti,ab,tw. (183)

38	(bias adj3 racial).ti,ab,tw. (441)

39	(bigotry or bigot*).ti,ab,tw. (157)

40	exp colonialism/ (119)

41	colonial*.ti,ab,tw. (5535)

42	exp desegregation/ (12)

43	exp health disparity/ (14602)

44	exp social segregation/ (108)

45	exp prejudice/ [includes racism, but also identifies other forms of discrimination] (2011)

46	(religious hatred or religious intolerance).ti,ab,tw. (11)

47	social marginali*.ti,ab,tw. (239)

48	exp social stigma/ (5668)

49	exp social desirability/ (4525)

50	exp stereotyping/ (1526)

51	or/31-49 (34005)

52	("People of color" or "people of colour").ti,ab,tw. [individuals experiencing discrimination] (414)

53	exp Black person/ (88882)

54	african continental ancestry group.mp. (69)

55	American Indian/ (16902)

56	american native continental ancestry.mp. (10)

57	(metis or mestizo).ti,ab,tw. (1583)

58	exp Asian American/ or exp Asian/ (187127)

59	asian:.ti,ab,tw. (108857)

60	exp Hispanic/ (53160)

61	hispanic*.ti,ab,tw. (55374)

62	exp "Romani (people)"/ (337)

63	Roma*.ti,ab,tw. (26750)

64	(aboriginal or eskimo* or Inuit* or Inuk or Metis* or first nation* or indigenous Canadians or native Canadian or Native America or Maori* or pacific islander* or American indian* or Amerindian or native alaska* or Alaska native or native Hawaiian or Torres strait or on-reserve or off-reserve or tribal or autochton* or amerindian* or indigene*).ti,ab,tw. (38459)

65	arab*.mp. or persian*.ti,ab. [mp=title, abstract, heading word, drug trade name, original title, device manufacturer, drug manufacturer, device trade name, keyword, floating subheading word, candidate term word] (150139)

66	exp semitic people/ (13484)

67	52 or 53 or 54 or 55 or 56 or 57 or 58 or 59 or 60 or 61 or 62 or 63 or 64 or 65 or 66 (592383)

68	51 and 67 (6664)

69	30 or 51 or 68 (39381)

70	25 and 69 (5259)

71	exp cultural competence/ [interventions] (5173)

72	cultural competenc:.ti,ab,tw. (3135)

73	Culturally Competent Care.ti,ab,tw. (549)

74	exp cultural diversity/ (771)

75	exp capacity building/ (3438)

76	exp community participation/ (876)

77	(community engag* or community particip* or community involv*).ti,ab,tw. (8831)

78	exp continuing education/ (30395)

79	exp evaluation study/ (48204)

80	exp practice guideline/ (450654)

81	guideline.ti,ab,tw. (67863)

82	exp health services research/ (31493)

83	exp in service training/ (15621)

84	intervention.ti,ab,tw. (696037)

85	intersectionality.mp. (385)

86	knowledge transfer.ti,ab,tw. (1593)

87	exp leadership/ (54923)

88	exp needs assessment/ (21315)

89	exp personnel management/ (85921)

90	((professional or staff) adj2 development).ti,ab,tw. (11955)

91	racism awareness.ti,ab,tw. (3)

92	exp program evaluation/ (16473)

93	(policy or polic*).ti,ab,tw. (275952)

94	((policy or practice) adj5 equitable).ti,ab,tw. (172)

95	equity research.mp. (35)

96	exp total quality management/ (48414)

97	quality improvement.ti,ab,tw. (39179)

98	or/71-97 (1690732)

99	70 and 98 (1845)

100	limit 99 to yr="2000 -Current" (1813)

101	limit 100 to (editorial or erratum or letter or note) (73)

102	100 not 101 (1740)

103	remove duplicates from 102 (1696)

***************************

**Database: PsycINFO <1806 to April Week 2 2018>**

**Search Strategy:**

--------------------------------------------------------------------------------

1	ambulatory care.ti,ab. (984)

2	exp Outpatient Treatment/ (6204)

3	exp Health Personnel Attitudes/ (21501)

4	exp Health Care Services/ and *Indigenous Populations/ (253)

5	exp Health Knowledge/ or exp Health Education/ or exp Health Behavior/ or exp Health Attitudes/ (53901)

6	exp Primary Health Care/ or exp Family Medicine/ or exp Family Physicians/ (18795)

7	(family adj2 (health or practice)).ti,ab. (6989)

8	outpatient.ti,ab. (31568)

9	exp Health Disparities/ (6572)

10	exp Urban Environments/ and exp Health Care Services/ (931)

11	exp Primary Health Care/ (16585)

12	exp Health Care Utilization/ or exp Health Care Delivery/ (48771)

13	((access adj5 healthcare).ti,ab. or access.mp.) adj5 healthcare.ti,ab. [mp=title, abstract, heading word, table of contents, key concepts, original title, tests & measures] (3731)

14	patient acceptance of health care.ti,ab. (0)

15	clinic*.ti,ab. (554349)

16	exp Community Mental Health Centers/ (2722)

17	exp Community Mental Health Centers/ (2722)

18	maternal-child health centers.mp. (3)

19	exp Drug Rehabilitation/ and exp Comorbidity/ (745)

20	walkin.ti,ab. (3)

21	walk-in.ti,ab. (681)

22	or/1-21 (687257)

23	exp RACISM/ (6936)

24	racism.ti,ab. (9367)

25	exp COGNITIVE BIAS/ or exp CULTURAL TEST BIAS/ (4182)

26	(bias adj3 class*).ti,ab. (348)

27	(bias adj3 implicit).ti,ab. (653)

28	(bias adj3 ethnic).ti,ab. (224)

29	(bias adj3 race).ti,ab. (339)

30	(bias adj3 racial).ti,ab. (888)

31	(bias adj3 social).ti,ab. (1368)

32	anti-indigenous.ti,ab. (3)

33	apartheid.ti,ab. (1244)

34	colonial*.ti,ab. (4986)

35	exp Social Integration/ (5123)

36	(desegregation or segregation).ti,ab. (7705)

37	exp PREJUDICE/ (7242)

38	exp "Racial and Ethnic Relations"/ (3379)

39	exp Social Identity/ or exp Intergroup Dynamics/ or exp Ingroup Outgroup/ (22164)

40	exp MARGINALIZATION/ (1901)

41	exp STIGMA/ (10277)

42	exp Social Desirability/ (2756)

43	exp Stereotyped Attitudes/ (11860)

44	Stereotyp*.mp. or prejudice*.ti,ab. (51008)

45	(white supremac* or white power).ti,ab. (422)

46	or/23-25 (15636)

47	exp Minority Groups/ (13660)

48	exp religious groups/ (11364)

49	exp Minority Groups/ or exp "Racial and Ethnic Groups"/ or exp "Racial and Ethnic Differences"/ (141205)

50	"people of color".ti,ab. (1298)

51	"People of colour".ti,ab. (33)

52	visible minorit*.ti,ab. (198)

53	exp Blacks/ (47853)

54	african continental ancestry group.mp. (2)

55	american native continental ancestry group.mp. (2)

56	asian continental ancestry group.mp. (6)

57	oceanic ancestry group.mp. (2)

58	diversity.ti,ab. (41175)

59	exp "Latinos/Latinas"/ (26056)

60	hispanic.ti,ab. (20279)

61	exp Romanies/ (269)

62	exp Romanies/ (269)

63	roma.ti,ab. (463)

64	exp Indigenous Populations/ (12390)

65	aboriginal.ti,ab. (3213)

66	(aboriginal or eskimo* or Inuit* or Inuk or Metis* or first nation* or indigenous Canadians or native Canadian or Native America or Maori* or pacific inslander* or American indian* or Amerindian or native alaska* or Alaska native or native Hawaiian or Torres strait or on-reserve or off-reserve or tribal or autochton* or amerindian* or indigene*).ti,ab. (13228)

67	exp American Indians/ or exp Alaska Natives/ (6495)

68	exp Asians/ (22124)

69	Asian*.ti. (7175)

70	african.af. (203376)

71	African American*.ti,ab. (42349)

72	Afro*.ti,ab. (1926)

73	exp ARABS/ (2748)

74	arab*.ti,ab. (9724)

75	or/47-74 (352003)

76	exp Cultural Sensitivity/ (6309)

77	cultural competence.ti,ab. (2568)

78	exp Cross Cultural Treatment/ (3606)

79	Culturally Competent Care.mp. (356)

80	*Diversity/ (4217)

81	*Continuing Education/ and exp Medical Education/ (206)

82	exp Program Evaluation/ or evaluation studies as topic.mp. (19297)

83	guideline.ti,ab. (5102)

84	exp TREATMENT GUIDELINES/ (6071)

85	guideline.ti,ab. (5102)

86	Health Services Research.mp. (1280)

87	Health Services Research.mp. (1280)

88	exp Inservice Training/ (3059)

89	Intervention.ti,ab. (201231)

90	exp Needs Assessment/ (3971)

91	exp Professional Development/ or Staff Development.mp. (18737)

92	((professional or staff) adj2 development).af. (49643)

93	racism awareness.mp. (29)

94	(policy or polic*).ti,ab. (164689)

95	program*.ti,ab. (357346)

96	Quality Control/ or Quality Improvement.mp. (5457)

97	exp Leadership/ (38797)

98	capacity building.mp. (1748)

99	exp Knowledge Transfer/ (2622)

100	Intersectionality.mp. (1777)

101	exp Community Involvement/ (4492)

102	Community engagement.ti,ab. (1180)

103	exp Policy Making/ (67067)

104	exp "EQUITY (SOCIAL)"/ (3314)

105	or/76-104 (747291)

106	22 and 46 and 75 and 105 (306)

***************************

Interdisciplinary Databases

**Scopus: Limited to 2000-2018**, articles only

554

## **(** **TITLE-ABS-KEY** **(** **racism** **)**  **OR**  **TITLE-ABS-KEY** **(** **racial**  AND **discrimination** **)**  **AND**  **TITLE-ABS-KEY** **(** **health**  AND **care**  AND **worker** **)**  **OR**  **TITLE-ABS-KEY** **(** **physician** **)** **OR**  **TITLE-ABS-KEY** **(** **health**  AND **personnel** **)**  **OR**  **TITLE-ABS-KEY** **(** **healthcare**  AND **worker** **)**  **AND**  **TITLE-ABS-KEY** **(** **staff**  AND **development** **)**  **OR**  **TITLE-ABS-KEY** **(** **in**  AND **service** **)**  **OR**  **TITLE-ABS-KEY** **(** **continuing**  AND **education** **)**  **AND**  **TITLE-ABS-KEY** **(** **quality**  AND **improvement** **)**  **OR**  **TITLE-ABS-KEY** **(** **health**  AND **equity** **)**  **OR**  **TITLE-ABS-KEY** **(** **racism**  AND **awareness** **)** **)**

**ERIC 57**

[(MAINSUBJECT.EXACT("Mental Health Workers") OR MAINSUBJECT.EXACT("Psychologists") OR MAINSUBJECT.EXACT("Health Facilities") OR MAINSUBJECT.EXACT("Allied Health Personnel") OR MAINSUBJECT.EXACT("Physicians") OR MAINSUBJECT.EXACT("Health Occupations") OR MAINSUBJECT.EXACT("Nurses") OR MAINSUBJECT.EXACT("Physician Patient Relationship")) AND (MAINSUBJECT.EXACT("Racism") OR (MAINSUBJECT.EXACT("Racial Factors") OR MAINSUBJECT.EXACT("Racial Bias") OR MAINSUBJECT.EXACT("Racial Discrimination") OR MAINSUBJECT.EXACT("Racism") OR MAINSUBJECT.EXACT("Racial Attitudes"))) AND (MAINSUBJECT.EXACT("Therapy") OR MAINSUBJECT.EXACT("Counseling") OR MAINSUBJECT.EXACT("Resource Room Programs") OR MAINSUBJECT.EXACT("Outreach Programs") OR MAINSUBJECT.EXACT("Human Services") OR MAINSUBJECT.EXACT("Consultation Programs") OR MAINSUBJECT.EXACT("Behavior Modification") OR MAINSUBJECT.EXACT("coeducational Methods") OR MAINSUBJECT.EXACT("Cognitive Restructuring") OR MAINSUBJECT.EXACT("Psychology")) OR (MAINSUBJECT.EXACT("Staff Role") OR MAINSUBJECT.EXACT("Staff Development") OR MAINSUBJECT.EXACT("Staff Orientation"))](https://search-proquest-com.myaccess.library.utoronto.ca/results.displayspellingsuggestions:dospellingsearch?t:ac=16EE58183C84450CPQ/1)

**Proquest via University of Toronto 280** (120 databases)

### 325

TOPIC: (Racism OR racial discrimination OR Implicit Bias OR Racial Bias) *AND* TOPIC:(health care outpatient OR healthcare OR health Care OR Doctor* OR physician* OR nurses OR health personnel) *AND* TOPIC: (equity OR intervention OR education OR quality improve* OR awareness OR staff development OR guideline OR evaluation Study OR program evaluation)

Refined by: DOCUMENT TYPES: ( ARTICLE ) AND WEB OF SCIENCE CATEGORIES: ( HEALTH CARE SCIENCES SERVICES OR NURSING OR MEDICINE GENERAL INTERNAL OR HEALTH POLICY SERVICES OR ETHICS OR MEDICAL INFORMATICS OR MEDICAL ETHICS OR MULTIDISCIPLINARY SCIENCES )

Timespan: 2000-2018. Indexes: SCI-EXPANDED, SSCI.
